# Analytical Agreement Between the Vision C Automated ESR Analyzer and the Manual Westergren Reference Method: A Prospective Method Comparison Study

**DOI:** 10.3390/diagnostics16152323

**Published:** 2026-07-24

**Authors:** Ayça Tuzcu, Kenan Yörük, Yusuf Kurtulmuş, Mustafa Yılmaz

**Affiliations:** Department of Biochemistry, Faculty of Medicine, Aydin Adnan Menderes University, 09100 Aydin, Türkiye; atuzcu@adu.edu.tr (A.T.); kenan-1997@hotmail.com (K.Y.); yusuf.kurtulmus@adu.edu.tr (Y.K.)

**Keywords:** erythrocyte sedimentation rate, method comparison, Westergren method, automated analyzer, Bland–Altman analysis, analytical bias

## Abstract

**Background/Objectives**: The erythrocyte sedimentation rate (ESR) is a widely used biomarker of systemic inflammation, with established roles in rheumatologic, infectious, and malignant disease. The Westergren method remains the reference procedure, but automated analyzers are increasingly used clinically, and methodological differences may introduce systematic bias. This study evaluated analytical agreement between the Vision C automated ESR analyzer (EDTA) and the Westergren reference (sodium citrate). **Methods**: This prospective, single-center study followed CLSI EP09-ED3. A total of 228 consecutive adult patients were enrolled. Agreement was assessed by Bland–Altman analysis, Passing–Bablok regression, and Pearson correlation, with subgroup analyses (<40, 40–80, >80 mm/h) and against RCPA allowable total error (TEa) specifications. **Results**: Bland–Altman analysis showed a significant mean bias of −3.30 mm/h (95% CI: −4.25 to −2.35), with increasing dispersion at higher ESR values indicating heteroscedasticity; regression-based limits of agreement therefore widened progressively with ESR magnitude. Passing–Bablok regression showed no proportional bias but a significant constant bias; subgroup regression was not performed due to truncated ranges. Correlation was strong overall (r = 0.961, *p* < 0.001) and declined across subgroups (r = 0.821, 0.740, 0.687), consistent with range restriction rather than reduced agreement. The analyzer met RCPA TEa criteria across both ranges (≤20 mm/h: bias −2.55 mm/h within ±6.0 mm/h; >20 mm/h: percentage bias −10.49% within ±30%), including the high-ESR subgroup (approximately 6–9%). **Conclusions**: The Vision C analyzer showed a significant, level-dependent negative bias versus Westergren, widening at higher ESRs; strong correlation and RCPA TEa compliance across the full range, including the high subgroup, support its suitability for routine use. Wide regression-based limits and the limited high subgroup sample size (*n* = 20) warrant caution and further study but do not support routine manual confirmation, since predefined acceptance criteria are met. Method-specific reporting and consistent instrument use in follow-up are recommended.

## 1. Introduction

The erythrocyte sedimentation rate (ESR) is one of the oldest and most widely used laboratory parameters for the assessment of systemic inflammation, reflecting the degree of acute-phase protein-driven erythrocyte aggregation in plasma [1]. Despite its lack of disease specificity—a limitation that has recently prompted calls for more selective, diagnostic-stewardship-guided use of ESR in infectious disease work-up [2]—the ESR remains an integral biomarker in the diagnosis and monitoring of a broad spectrum of inflammatory conditions, including infectious, malignant, and autoimmune disorders [3,4], retaining specific, non-redundant clinical utility in the contexts outlined below. Its clinical relevance is particularly pronounced in rheumatology, where the ESR is incorporated into the 2010 ACR/EULAR classification criteria for rheumatoid arthritis (RA) [5] and serves as a core component of the Disease Activity Score 28 (DAS28)—a validated composite index widely used to assess disease activity and guide therapeutic decision-making [6]. The ESR also plays a pivotal role in the diagnosis and activity monitoring of large-vessel vasculitis and polymyalgia rheumatica, conditions in which ESR elevation carries direct implications for treatment initiation and dose adjustment [7,8]. Given its integration into internationally validated disease activity indices and clinical decision algorithms, the analytical accuracy and between-method comparability of ESR measurements carry direct implications for patient management.

The manual Westergren method, performed with sodium citrate-anticoagulated samples, is currently recommended as the reference procedure for ESR measurement by the International Council for Standardization in Haematology (ICSH) [1]. Despite its longstanding standardization, the Westergren method presents several practical limitations in high-volume clinical laboratories, including manual operation, prolonged turnaround time, operator dependency, open-tube handling with associated biohazard risks, and susceptibility to preanalytical variables [9]. These limitations have driven the widespread adoption of automated ESR analyzers, which offer shorter turnaround times, closed-tube sampling, seamless integration into laboratory workflows, and reduced operator variability. However, automated systems often employ EDTA-anticoagulated samples and differ from the reference method in their measurement principles and computational algorithms, precluding a direct assumption of equivalence with Westergren-based results [9]. Consequently, any automated ESR system introduced into clinical practice requires rigorous method comparison against the Westergren reference to establish analytical agreement and to characterize the nature and magnitude of potential systematic or proportional bias.

Several studies have evaluated the analytical agreement between automated ESR analyzers and the Westergren reference method, reporting variable degrees of concordance and differing magnitudes of systematic bias, particularly at elevated ESR values [10,11]. Discrepancies between methods have been attributed to differences in anticoagulant type, measurement principles, and correction algorithms applied by automated systems [9,10]. These methodological differences are of particular clinical relevance, as ESR values obtained by different methods may not be directly interchangeable, potentially leading to misclassification of disease activity or inappropriate treatment decisions when transitioning between measurement system [12]. Notably, limited data exist on the analytical performance of the Vision automated ESR analyzer in direct comparison with the Westergren method, highlighting the need for a rigorous, guideline-based method comparison study.

The present study was designed as a prospective method comparison study conducted in accordance with the CLSI EP09-ED3 guideline [12]. The primary aim was to evaluate the analytical agreement between ESR values obtained by the Vision automated analyzer using EDTA-anticoagulated samples and those measured by the manual Westergren reference method using sodium citrate-anticoagulated samples, across a clinically representative range of ESR values encompassing low, intermediate, and high measurement categories (*n* = 228). Secondary objectives included the assessment of fixed and proportional bias and the identification of clinically relevant discordance at established decision thresholds.

## 2. Materials and Methods

### 2.1. Study Design and Setting

This prospective, single-center method comparison study was conducted in the Clinical Biochemistry Laboratory of Aydın Adnan Menderes University Hospital between March 2026 and May 2026. The study protocol was approved by the Non-Interventional Clinical Research Ethics Committee of Aydın Adnan Menderes University, Faculty of Medicine (Decision No: 2026/79; Date: 16 February 2026). Informed consent was waived due to the observational nature of the study. It should be noted that, while the EDTA sample used for Vision C (Shenzhen YHLO Biotech Co., Ltd., Shenzhen, China) measurement corresponded to the tube already collected for routine clinical ESR testing, the sodium citrate sample required for the manual Westergren reference method was collected specifically for study purposes and does not, strictly speaking, constitute a residual sample from routine care. This additional tube was drawn during the same venipuncture session as the routine sample, without a separate needle stick, and the associated waiver of separate written consent was reviewed and approved by the same Ethics Committee as part of the study protocol, on the basis of the minimal additional blood volume (approximately 1.6 mL) and the absence of any additional procedural risk beyond the routine venipuncture. We acknowledge this distinction for full methodological transparency. All patient data were anonymized prior to analysis; each sample was assigned a sequential study identification number, and no patient names or other direct identifiers were used or retained in the study database. The study was designed in accordance with the CLSI EP09-ED3 guideline for measurement procedure comparison and bias estimation [12], and ESR measurements were performed in compliance with ICSH recommendations for a standardized ESR methodology [1].

### 2.2. Study Population and Sample Collection

Adult patients (aged ≥18 years) for whom ESR testing was requested as part of routine clinical evaluation were prospectively enrolled using a consecutive sampling approach. A priori sample size estimation was performed using G*Powerv3.1.9.7 software based on Pearson correlation analysis, with a significance level of α = 0.05 and a power of 0.80, which indicated a minimum sample size of 90. A total of 228 samples were collected and analyzed, exceeding the minimum requirement and enhancing the statistical power of the study. We acknowledge, however, that Pearson correlation power is not the primary determinant of adequacy in a method-comparison (agreement) study; per the CLSI EP09-ED3 guide-line, the more relevant metric is the precision of the estimated bias and limits of agreement, reflected in the width of their 95% confidence intervals [12]. Post hoc, the achieved precision for mean bias was ±0.95 mm/h for the overall sample (*n* = 228), ±0.78 mm/h for the low-ESR subgroup (*n* = 172), ±3.60 mm/h for the intermediate subgroup (*n* = 36), and ±5.32 mm/h for the high-ESR subgroup (*n* = 20). This confirms that, while the overall and low ESR bias estimates were determined with good precision, estimates in the intermediate and especially the high-ESR subgroup carry considerably wider confidence intervals, and conclusions drawn from this subgroup should be interpreted with corresponding caution.

Blood samples were obtained during a single venipuncture session. For automated ESR measurement, blood was collected into 3 mL K_2_-EDTA tubes (BD Vacutainer, Becton Dickinson, Franklin Lakes, NJ, USA). Simultaneously, without an additional venipuncture, a sodium citrate-anticoagulated sample (Vacuplus sedimentation tubes, Rapida Medikal, Ankara, Turkey; 8 × 120 mm, 1.6 mL; sodium citrate 0.105 M, blood-to-citrate ratio 1:4) was collected for manual Westergren measurement. All measurements were performed within two hours of sample collection under standardized laboratory conditions.

Samples were excluded if any of the following criteria were met: (i) visible clot formation, (ii) insufficient sample volume, (iii) visible hemolysis, or (iv) preanalytical delay exceeding two hours. To ensure adequate representation across the clinically relevant ESR measurement range, samples were categorized into three subgroups: low (ESR < 40 mm/h, *n* = 172), intermediate (ESR 40–80 mm/h, *n* = 36), and high (ESR > 80 mm/h, *n* = 20), yielding a total of 228 analyzable samples.

### 2.3. Automated ESR Measurement

Automated ESR measurements were performed using the Vision C ESR Analyzer (YHLO Biotech Co., Ltd., Shenzhen, China) [10]. The analyzer employs an infrared optical detection system and uses primary K_2_-EDTA-anticoagulated whole blood collected in standard tubes (12–13 mm diameter). Prior to analysis, samples are automatically mixed by 180° rotation. Erythrocyte sedimentation is assessed using an infrared emitter–receiver scanning system with a reported scanning resolution of 0.25 mm. The minimum required sample volume is 1.5 mL, the analytical measurement range is 0–120 mm/h, and each analytical cycle is completed within 20 min. Results are reported as the equivalent 60 min Westergren value.

Daily internal quality control (IQC) was performed using manufacturer-provided ready-to-use control tubes containing EDTA and reference ball bearings, available at two distinct control levels as part of the laboratory’s routine quality assurance procedures. The Vision C analyzer therefore operates under a two-level internal quality control scheme, consistent with the minimum requirements of ISO 15189 [13] for internal quality control. The analyzer does not require routine user calibration, as instrument calibration is factory-set by the manufacturer; no additional laboratory calibration was performed during the study period. External quality assessment for ESR was conducted through participation in the American Association of Bioanalysts Molecular Laboratory Evaluation (AAB MLE) program, which provides an ESR-specific proficiency testing module and the laboratory maintained acceptable performance throughout the study period.

The Vision C analyzer does not apply an automatic internal hematocrit (HCT) correction to the reported ESR value. Per the manufacturer’s operation manual, when sample HCT is below 35%, ESR results are recommended to be calibrated according to HCT; however, this manual calibration step was not applied to the ESR results used in the present study, and reported Vision C values therefore reflect uncorrected instrument output. Patient HCT values, obtained from concurrent complete blood count testing, were available for all study participants but were not used to adjust the reported ESR results or incorporated into the present analysis; the potential influence of hematocrit on inter-method agreement is acknowledged as a limitation in the Discussion.

### 2.4. Reference Westergren Method

Manual ESR measurements were performed using the reference Westergren method in accordance with ICSH recommendations [1]. Sodium citrate-anticoagulated samples were collected into Vacuplus sedimentation tubes (Rapida Medikal, Ankara, Turkey; 8 × 120 mm, 1.6 mL; sodium citrate 0.105 M, blood-to-citrate ratio 1:4). Samples were placed vertically in a standard Westergren stand on a vibration-free surface at a controlled room temperature of 22 °C, and the sedimentation distance was read at 60 min by a single trained laboratory technician. Results were recorded in mm/h.

### 2.5. Statistical Analysis

Statistical analyses were performed in accordance with the CLSI EP09-ED3 guideline [12] and ICSH recommendations [1], using the Analyse-it statistical software v6.16.2 (Analyse-it Software, Ltd., Leeds, UK) as an Excel add-in. Descriptive statistics were reported as median and interquartile range (IQR), with minimum and maximum values, given the non-parametric distribution of ESR data.

Analytical agreement between the Vision C automated analyzer and the manual Westergren reference method was evaluated using Pearson correlation analysis, Passing–Bablok regression [14], and Bland–Altman difference plots [15]. The Pearson correlation coefficient (r) was calculated to assess the strength of the linear relationship between methods. Passing–Bablok regression was applied to the full study sample only (*n* = 228); within-subgroup regression analysis was not performed, as the method requires an adequate spread of values across the measurement range and application within narrow, truncated ESR ranges yields extrapolation artefacts rather than informative bias estimates [15]. Where applied, a slope confidence interval including 1 indicated absence of proportional bias, and an intercept confidence interval including 0 indicated absence of constant bias. Bland–Altman analysis was performed with differences calculated as Vision C minus Westergren (mm/h), and mean bias with 95% limits of agreement (LoA) were determined across the full measurement range and within each ESR subgroup.

Subgroup analyses were performed according to ESR measurement categories—low (<40 mm/h), intermediate (40–80 mm/h), and high (>80 mm/h)—in accordance with ICSH recommendations, to evaluate analytical performance across clinically relevant decision thresholds [1]. Method performance was further assessed against the allowable total error (TEa) criteria defined by the Royal College of Pathologists of Australasia (RCPA) Haematology Analytical Performance Specifications: for ESR values ≤ 20 mm/h, an absolute bias limit of ±6.0 mm/h was applied, and for values > 20 mm/h, a percentage bias limit of ±30% was used [16]. Statistical significance was accepted at *p* < 0.05.

### 2.6. Use of Generative Artificial Intelligence

During the preparation of this manuscript, the authors used Claude Opus 4.6 (Anthropic, San Francisco, CA, USA) to assist with code review for statistical analyses, to improve the fluency of the English text, and to format data tables. No generative AI tool was used in the study design, data collection, data analysis, or interpretation of results. All AI-assisted output was reviewed and edited by the authors, who take full responsibility for the content of this publication.

## 3. Results

### 3.1. Study Population

A total of 228 consecutive adult patients were included in the study. Of these, 138 (60.5%) were female and 90 (39.5%) were male. The median age was 56 years (IQR: 43.8–65.0; range: 18–84 years). Vision C ESR values ranged from 5 to 115 mm/h (median: 15, IQR: 10.0–35.0) and Westergren ESR values ranged from 2 to 119 mm/h (median: 11, IQR: 4.0–32.2). The majority of measurements fell in the low ESR range: 172 samples (75.4%) had ESR values below 40 mm/h, 36 (15.8%) between 40–80 mm/h, and 20 (8.8%) above 80 mm/h. Descriptive characteristics of the study population are summarised in Table 1.

### 3.2. Overall Method Comparison

Analytical agreement between the Vision C automated analyser and the Westergren reference method was evaluated across the full study sample (*n* = 228). Pearson correlation analysis demonstrated a strong linear association between the two methods (r = 0.961, *p* < 0.001). Passing–Bablok regression yielded the equation Vision C = −2.857 + 0.971 × Westergren. The 95% CI for the slope (0.922–1.014) included 1, indicating no proportional bias, whereas the 95% CI for the intercept (−3.167 to −1.714) excluded 0, indicating a statistically significant constant bias.

In the conventional Bland–Altman analysis, the mean difference (Vision C minus Westergren) was −3.30 mm/h (95% CI: −4.25 to −2.35 mm/h), confirming a statistically significant systematic negative bias between the two methods. However, the difference plot showed increasing dispersion at higher ESR values, and regressing absolute residuals on Westergren ESR confirmed this heteroscedasticity formally (r = 0.49, *p* < 0.001). This pattern is consistent with recent methodological work showing that classical Bland–Altman limits of agreement become unreliable under non-constant bias and variance, and that regression of the differences against the reference method, or fractional-polynomial modelling of the difference and residual variance, provides a more valid representation of agreement across the measurement range [17,18]. The conventional whole-cohort limits of agreement were therefore considered descriptive only and were not used as the primary measure of agreement across the full measurement range.

To account for the non-constant bias and variance, regression-based limits of agreement were calculated using Westergren ESR as the predictor. The bias equation was

Difference = −2.235 − 0.0394 × Westergren

where Difference represents Vision C minus Westergren. The variability of residuals also increased with ESR magnitude and was modeled as

|residual| = 2.559 − 0.0958 × Westergren

**Figure 1:**
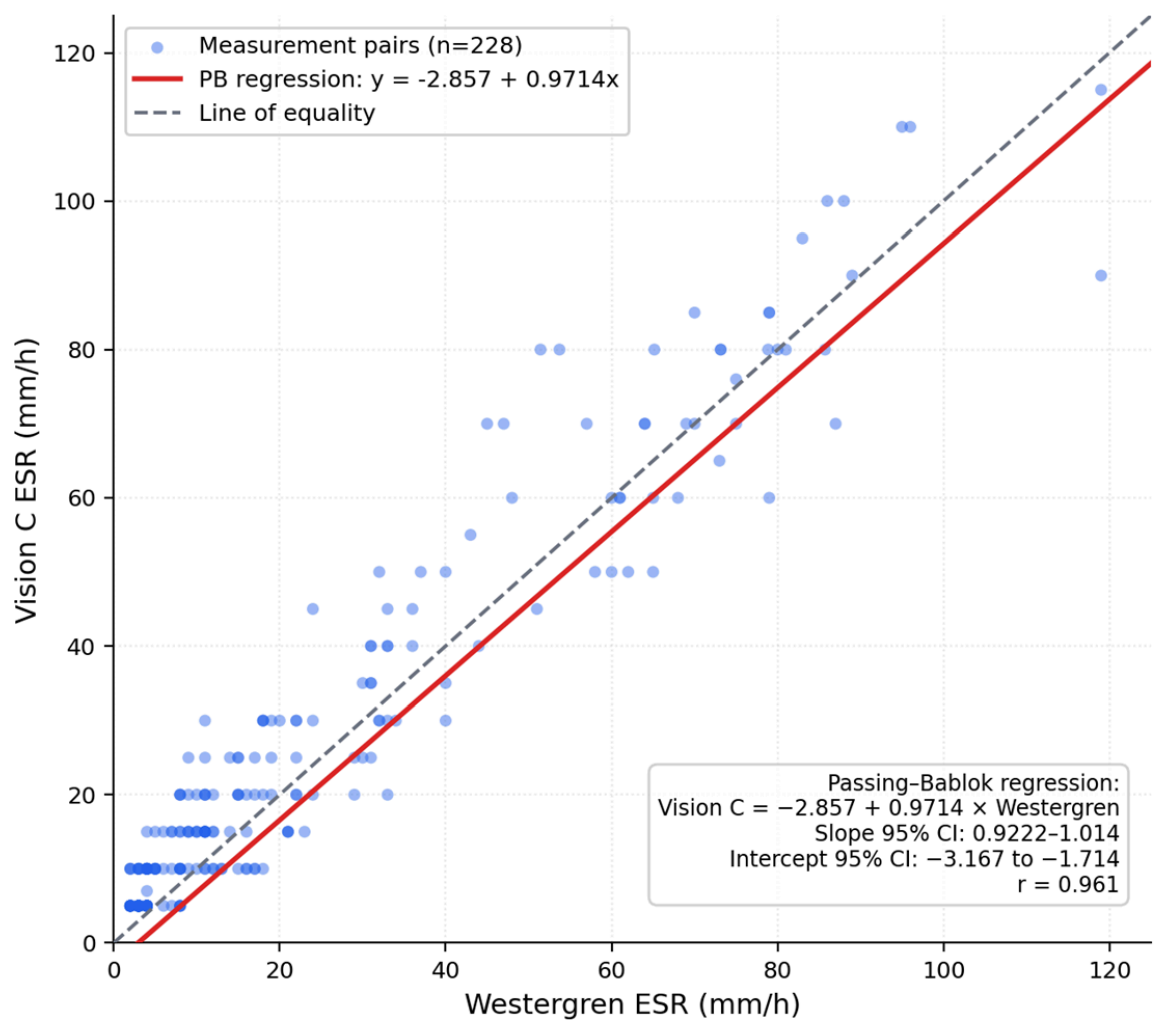
Passing–Bablok regression analysis comparing Vision C and Westergren ESR measurements (*n* = 228). The solid line represents the regression line (Vision C = −2.857 + 0.971 × Westergren); the dashed line represents the line of equality. The slope 95% CI (0.922–1.014) includes 1, indicating no proportional bias. The intercept 95% CI (−3.167 to −1.714) excludes 0, indicating a statistically significant constant bias (r = 0.961).

**Figure 2:**
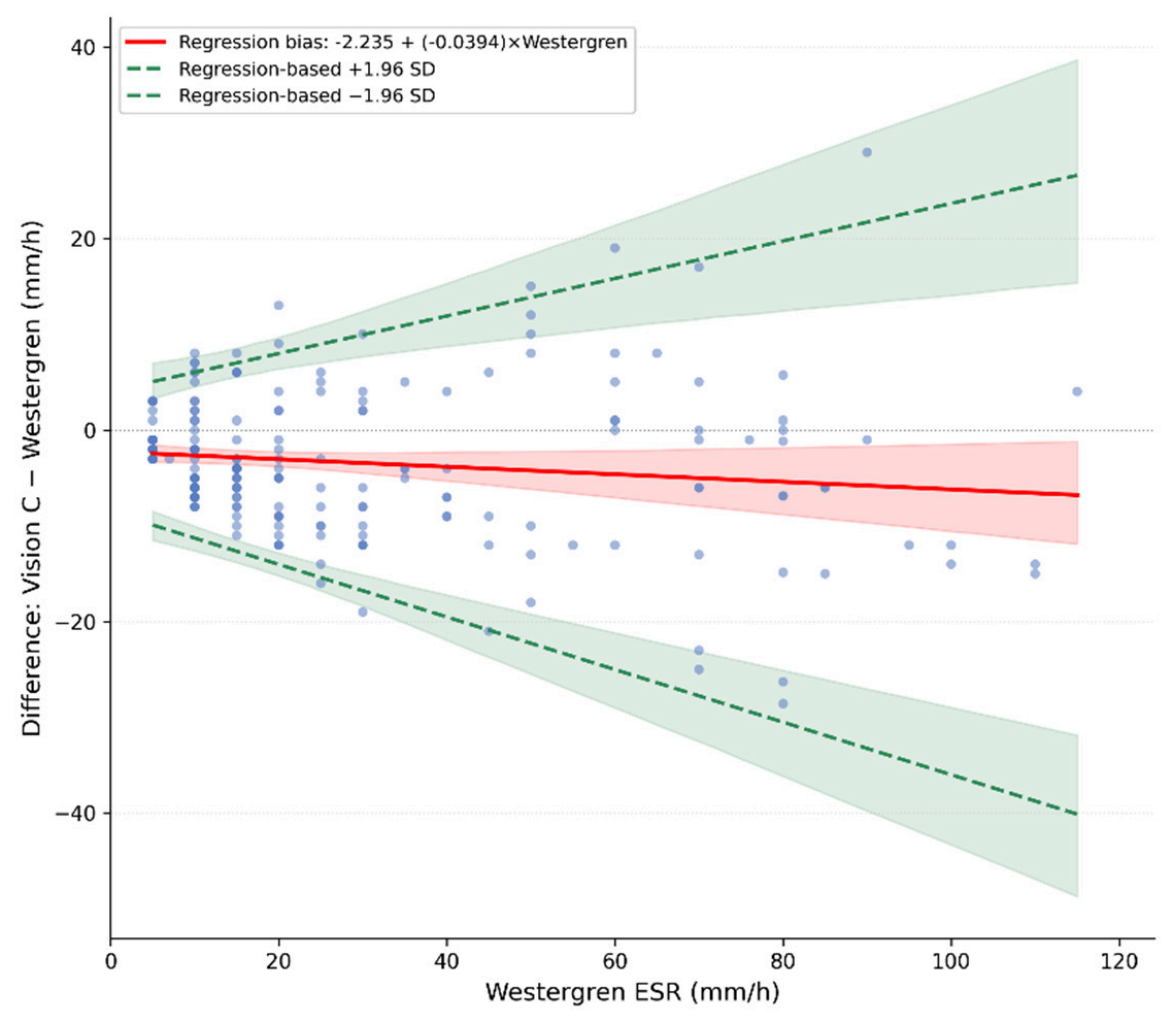
Bland–Altman difference plot for the overall sample (*n* = 228). Differences are expressed as Vision C minus Westergren (mm/h). Increasing dispersion at higher ESR values indicated heteroscedasticity, confirmed by regressing absolute residuals on Westergren ESR (r = 0.49, *p* < 0.001). Regression-based limits of agreement were therefore calculated per CLSI EP09-ED3. The red line shows the regression-based bias (Difference = −2.24 − 0.039 × Westergren, *p* = 0.033); green dashed lines show the corresponding ±1.96 SD limits, both with 95% confidence bands (bootstrap, 2000 resamples). Agreement limits widen with increasing ESR (e.g., −9.9 to 5.0 mm/h at 5 mm/h vs. −40.1 to 26.6 mm/h at 115 mm/h), showing that fixed limits misrepresent disagreement across the range. Data above ~80 mm/h are sparser; wide bands at the upper end should be interpreted accordingly.

Accordingly, the regression-based limits of agreement widened progressively across the measurement range: bias −3.02 mm/h (limits: −14.02 to 7.98 mm/h) at 20 mm/h, −3.81 mm/h (−19.51 to 11.89 mm/h) at 40 mm/h, −5.38 mm/h (−30.51 to 19.74 mm/h) at 80 mm/h, and −6.96 mm/h (−41.50 to 27.58 mm/h) at 120 mm/h. These findings indicate that agreement between the two methods is level-dependent, with greater variability at higher ESR values. Results are illustrated in Figure 1 and Figure 2.

### 3.3. Subgroup Analysis by ESR Level

**Figure 3:**
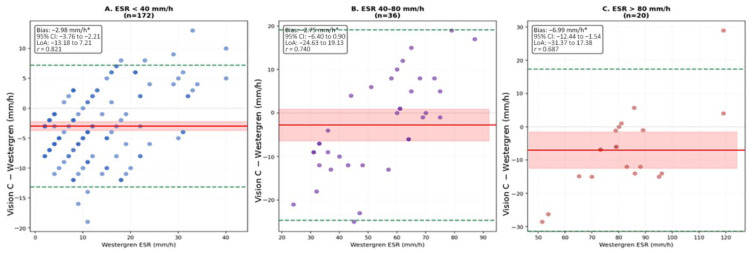
Bland–Altman difference plots by ESR subgroup. (**A**): ESR < 40 mm/h (*n* = 172); (**B**): ESR 40–80 mm/h (*n* = 36); (**C**): ESR > 80 mm/h (*n* = 20). Differences are expressed as Vision C minus Westergren (mm/h). Solid lines indicate mean bias; dashed lines indicate 95% limits of agreement (LoA); shaded areas represent the 95% CI of the mean bias. Asterisk (*) denotes statistically significant bias (95% CI excludes zero). A progressive increase in bias magnitude and widening of LoA is observed with increasing ESR levels.

Subgroup analyses were performed across three ESR categories in accordance with ICSH recommendations. Results are summarised in Table 2 and illustrated in Figure 3.

In the low-ESR subgroup (<40 mm/h, *n* = 172), the mean bias was −2.98 mm/h (95% CI: −3.76 to −2.21 mm/h), a statistically significant negative bias. The Passing–Bablok slope (1.0; 95% CI: 0.85–1.13) and intercept (−3.0; 95% CI: −3.6 to 1.25) both included their respective null values, indicating neither proportional nor constant bias in this range. The 95% LoA ranged from −13.15 to 7.18 mm/h, and the Pearson correlation was r = 0.821, reported descriptively.

In the intermediate-ESR subgroup (40–80 mm/h, *n* = 36), the mean bias was −2.75 mm/h (95% CI: −6.35 to 0.85 mm/h), not statistically significant. The 95% LoA widened considerably from −24.33 to 18.83 mm/h, reflecting greater inter-method variability at this range. The correlation weakened to r = 0.740; this decline relative to the overall sample likely reflects range restriction within the truncated subgroup rather than a genuine loss of agreement. Passing–Bablok regression was not performed within this subgroup, as the method requires an adequate spread of values, and its application to a truncated range produces extrapolation artefacts rather than meaningful bias estimates [15].

In the high-ESR subgroup (>80 mm/h, *n* = 20), the mean bias increased to −6.99 mm/h (95% CI: −12.31 to −1.68 mm/h), a statistically significant negative bias. This subgroup showed the widest 95% LoA (−30.75 to 16.77 mm/h) and the lowest correlation (r = 0.687) among the three strata, indicating markedly reduced agreement at elevated ESR values. Passing–Bablok regression was again omitted, as the small sample size and truncated range preclude meaningful regression-based bias estimation.

### 3.4. Assessment Against Allowable Total Error Criteria

To reconcile the clinical subgroup classification (<40, 40–80, and >80 mm/h) used in Section 3.1, Section 3.2 and Section 3.3 with the TEa-based threshold applied below, it should be noted that these two schemes are nested rather than independent. The ≤20 mm/h TEa stratum (*n* = 143) falls entirely within the <40 mm/h clinical subgroup, whereas the >20 mm/h TEa stratum (*n* = 85) comprises the remaining upper portion of the <40 mm/h subgroup (*n* = 29, ESR 20–40 mm/h) together with the entire 40–80 mm/h (*n* = 36) and >80 mm/h (*n* = 20) subgroups. Table 3 summarises this correspondence.

Method performance was assessed against the RCPA Haematology Analytical Performance Specifications (APS) [16]. For ESR values ≤ 20 mm/h (*n* = 143), where an absolute bias criterion applies, the mean bias was −2.55 mm/h, which fell within the ±6.0 mm/h TEa threshold. For ESR values > 20 mm/h (*n* = 85), where a percentage bias criterion applies, the mean percentage bias was −10.49%, which remained within the ±30% TEa limit. In both measurement ranges, the observed bias fell within the respective allowable error boundaries, indicating that the Vision C analyser meets the analytically acceptable performance criteria defined by RCPA across the full clinical ESR measurement range.

## 4. Discussion

The present study evaluated the analytical agreement between the Vision C automated ESR analyzer and the manual Westergren reference method across a clinically representative sample of 228 patients, encompassing low, intermediate, and high ESR measurement ranges. Overall, a strong linear correlation was observed between the two methods (r = 0.961), and Passing–Bablok regression analysis demonstrated the absence of proportional bias across the full measurement range. However, a statistically significant constant bias was identified, reflecting a systematic negative bias of −3.30 mm/h between the Vision C analyzer and the Westergren reference method. These findings indicate that while the two methods are strongly correlated, they are not fully interchangeable, and the nature of the observed bias warrants careful consideration in clinical practice.

The observed constant bias is consistent with the known methodological differences between automated EDTA-based ESR analyzers and the manual Westergren method using sodium citrate-anticoagulated samples. The Vision C analyzer employs an infrared optical scanning system to dynamically assess erythrocyte sedimentation kinetics in EDTA-anticoagulated whole blood, whereas the Westergren method relies on passive gravitational sedimentation measured at a fixed 60 min endpoint in a sodium citrate-anticoagulated sample. Differences in anticoagulant type, blood-to-anticoagulant ratio, tube geometry, and computational algorithms used to convert kinetic sedimentation data into equivalent Westergren values are recognized sources of systematic bias between automated and reference methods [1,9]. Importantly, these sources are confounded rather than separable in the present design: the observed bias reflects the combined, non-partitionable influence of the Vision C analyzer’s optical measurement principle, the use of EDTA rather than sodium citrate anticoagulation, the associated differences in blood-to-anticoagulant dilution ratio, and the proprietary algorithm extrapolating 20 min kinetic sedimentation data to an equivalent 60 min Westergren value. The present study design does not permit apportioning the relative contribution of each factor to the overall bias. The magnitude of the constant bias observed in the present study (−3.30 mm/h) is modest at low ESR levels and may not carry direct clinical significance in routine practice; the Bland–Altman difference plot demonstrated increasing dispersion of differences at higher ESR values, indicating that this average bias does not fully represent agreement across the entire measurement range. Therefore, it represents a systematic shift that clinicians and laboratory professionals should be aware of, particularly when interpreting serial ESR measurements obtained by different methods [19] or when applying ESR-based disease activity indices such as DAS28 [5].

Subgroup analyses revealed a progressive widening of limits of agreement and increasing bias magnitude with increasing ESR levels, a pattern with important clinical implications. Beyond this categorical subgroup analysis, regression-based limits of agreement modelling bias as a continuous function of Westergren ESR (Figure 2) confirmed this pattern directly: agreement limits narrowed at low ESR values and widened substantially at high values, with 95% confidence bands around both the bias and limit lines. This indicates that the fixed whole-cohort limits underestimate disagreement at the upper end of the measurement range while overstating it at the lower end—precisely at the ESR thresholds most relevant to clinical decision-making. In the low-ESR subgroup (<40 mm/h), the mean bias was modest, statistically significant, yet analytically acceptable; Passing–Bablok regression confirmed the absence of both proportional and constant bias in this range. In the intermediate subgroup (40–80 mm/h), the mean bias did not reach statistical significance, but the considerably widened limits of agreement (−24.33 to 18.83 mm/h) indicate increasing inter-method variability at this level. The Pearson correlation weakened to r = 0.740; this decline relative to the overall sample is attributable in part to range restriction within the truncated subgroup domain and should not be interpreted as independent evidence of reduced agreement. Within-subgroup Passing–Bablok regression was not applied, as regression within a truncated domain yields artefactual estimates that cannot be meaningfully interpreted as evidence of scale-dependent bias [15]; interpretation in this subgroup therefore relies on Bland–Altman-derived parameters alone. In the high-ESR subgroup (>80 mm/h), the mean bias increased to −6.99 mm/h, and the limits of agreement were the widest among all subgroups (−30.75 to 16.77 mm/h), providing the primary evidence of reduced inter-method concordance at elevated ESR values. As in the intermediate subgroup, the weakened correlation (r = 0.687) partly reflects range restriction as within-group variance narrows with subdivision, rather than a true deterioration in agreement; Passing–Bablok regression was omitted for the same methodological reasons. Taken together, these findings suggest that in patients with high ESRs—a clinical context frequently encountered in active vasculitis, polymyalgia rheumatica, and severe infectious or inflammatory states [7,8]—automated Vision C results should be interpreted with caution. The wide limits of agreement and the limited subgroup sample size (*n* = 20) introduce interpretive uncertainty at the individual-patient level, even though the method meets its predefined TEa acceptance criteria in this range.

The findings of the present study are broadly consistent with previously published data on the analytical performance of automated ESR analyzers. Şirin et al. [11], who evaluated the same Vision C analyzer against the Westergren reference method, reported an overall correlation coefficient of 0.948 and a mean bias of −5.23 mm/h, with subgroup-specific bias values of −0.885, −9.23, and −17.26 mm/h for the low, intermediate, and high-ESR subgroups, respectively. This pattern of progressively increasing bias at higher ESR levels closely mirrors the findings of the present study, in which subgroup-specific mean bias values were −2.98, −2.75, and −6.99 mm/h across the same ESR categories, and correlation coefficients similarly decreased from 0.821 to 0.687 with increasing ESR levels. Notably, the magnitude of bias observed in the present study was consistently lower than that reported by Şirin et al., which may reflect differences in study population characteristics, sample size, or preanalytical conditions. Walle et al. [10], evaluating a different automated ESR system against the Westergren method, likewise reported acceptable agreement at low ESR values but increasing inter-method variability at elevated ESR levels, further corroborating the observation that automated analyzers may show greater deviation from Westergren-assigned values relative to the Westergren reference at higher measurement ranges [11,20]. In contrast, Tomassetti et al. [9], in a large multicenter study (*n* = 787) evaluating three different automated ESR analyzers—VES-MATIC 5, CUBE 30 TOUCH, and MINI-CUBE—against the Westergren method, reported higher correlation coefficients (Spearman R^2^: 0.977–0.981) and substantially lower mean bias values (ranging from −2.1 to 2.5 mm/h), with all three analyzers demonstrating optimal accuracy, precision, and repeatability across the measurement range. Comparable performance has been reported for other modified-Westergren automated platforms: the VES-MATIC 5 system achieved a Passing–Bablok correlation of approximately 0.96 with the Westergren reference in routine practice [21], a dedicated evaluation published in this same journal similarly confirmed strong accuracy alongside 24–36 h sample stability for VES-MATIC 5 relative to the Westergren method [22], and the CUBE 30 touch analyzer has been independently validated against Westergren-derived reference intervals [23]. Consistent with these findings age- and sex-stratified reference intervals recently established for the VES-MATIC 5 and CUBE 30 touch platforms relative to the Westergren method have further confirmed close concordance across the clinically relevant measurement range, with only marginal (≈1 mm/h) deviations in mean and median values [24]. We note that Spearman R^2^—the squared rank-based (Spearman) correlation coefficient—is not statistically equivalent to the Pearson correlation coefficient (r) reported in the pre-sent study: the former is a non-parametric measure of monotonic association expressed on a squared, coefficient-of-determination scale, whereas the latter is a parametric measure of linear association reported directly. Direct numerical comparison between the two statis-tics is therefore not appropriate, and the higher values reported by Tomassetti et al. should not be interpreted as indicating stronger linear agreement than observed in the present study. Critically, all three analyzers in that study used sodium citrate anticoagulation, matching the Westergren reference, whereas the Vision C analyzer evaluated here uses EDTA. This contrast—near-zero bias for citrate-based automated analyzers versus the significant constant bias of −3.30 mm/h observed for the EDTA-based Vision C analyzer in the pre-sent study—constitutes, in our view, the most direct evidence available that anticoagulant mismatch, rather than an intrinsic inaccuracy of the automated optical measurement principle itself, is a dominant contributor to the bias observed in EDTA-based ESR analyzers. This finding underscores that analytical agreement in ESR method comparison is critically dependent on anticoagulant concordance with the reference method, in addition to and potentially outweighing analyzer-specific measurement principles and correction algorithms [25]. This interpretation is further supported by paired within-patient comparisons showing that ESR values measured directly in EDTA-anticoagulated blood differ systematically and significantly from hematocrit-adjusted, sodium citrate-based Westergren values obtained from the same individuals, independent of any automated measurement principle [26].

Assessment of method performance against the RCPA Haematology Analytical Performance Specifications revealed that the Vision C analyzer met the predefined allowable total error criteria across both measurement ranges evaluated. For ESR values ≤ 20 mm/h, the observed mean bias of −2.55 mm/h fell within the ±6.0 mm/h absolute TEa threshold, and for values > 20 mm/h, the mean percentage bias of −10.49% remained well within the ±30% TEa limit [16]. It should be noted, however, that TEa thresholds are not universally standardized and can vary substantially depending on whether they are derived from biological variation, regulatory (e.g., CLIA), or peer-group specifications, with the same analyte occasionally classified as acceptable under one framework and unacceptable under another [27]; this underscores the importance of explicitly specifying RCPA-based criteria, as done in the present study, when reporting TEa-based conclusions. These findings suggest that, at the level of overall analytical performance, the Vision C analyzer satisfies the minimum quality requirements specified for clinical ESR measurement. However, it is important to note that TEa-based assessment reflects aggregate performance across a broad measurement range and may not capture the full extent of inter-method disagreement at specific clinical decision points. As demonstrated by the subgroup analyses, the limits of agreement widened substantially at intermediate and high ESR levels. In the high-ESR subgroup, where the applicable RCPA criterion is the percentage-bias threshold (±30%) rather than the absolute threshold, the observed mean bias of −6.99 mm/h corresponds to a percentage bias of approximately 6–9% relative to the subgroup’s Westergren values—comfortably within the ±30% limit and therefore still compliant with the analyzer’s own predefined acceptance criterion. Nonetheless, this margin is narrower in relative terms than that observed in the low ESR range, and the wide limits of agreement at this level indicate that individual-level discordance can be substantial even when aggregate TEa compliance is met. Global TEa compliance should therefore not be interpreted as uniform analytical equivalence across the entire measurement range, particularly for individual clinical decisions made at the high end of the scale. This distinction is particularly relevant in clinical contexts where ESR values are used to guide treatment decisions, such as in the management of large-vessel vasculitis and polymyalgia rheumatica, where even modest measurement discrepancies may have meaningful clinical consequences [7,8]. This concern is not merely theoretical: the 2022 ACR/EULAR classification criteria for giant cell arteritis assign a fixed weight of three points to an ESR value of 50 mm/h or greater, a threshold whose determination is inherently method-dependent and therefore directly sensitive to the type of inter-method bias characterized in the present study [28].

Whether the observed width of the limits of agreement is clinically acceptable de-pends on how ESR is used in the specific monitoring context. For DAS28, the ESR term enters the formula as 0.70 × ln(ESR); this logarithmic transformation substantially dampens the impact of absolute ESR discrepancies, particularly at higher ESR values. For example, at an ESR of approximately 100 mm/h, a shift in the magnitude observed in the high-ESR subgroup’s limits of agreement (−30.75 to +16.77 mm/h) would alter the DAS28 ESR term by approximately 0.11–0.26 points, well below the established minimal clinically important difference of 1.2 points for DAS28 [29]. In this context, therefore, the observed inter-method disagreement is unlikely to materially affect disease activity classification. This reassurance should nonetheless be interpreted cautiously, given that DAS28-ESR and DAS28-CRP themselves classify disease activity discordantly in a substantial proportion of patients even when both markers are measured without analytical error (Greenmyer et al., 2020), indicating that inter-method ESR variability of the magnitude reported here is unlikely to be the dominant source of disease-activity misclassification within this composite index [30]. In contrast, ESR-based monitoring of large-vessel vasculitis and polymyalgia rheumatica typically relies on the absolute ESR value or its relative change from baseline (e.g., to identify relapse or to guide glucocorticoid tapering), without a comparable dampening trans-formation; in this setting, a limits-of-agreement span of approximately 47 mm/h at high ESR levels could plausibly influence clinical interpretation, particularly when ESR values obtained by different methods are compared directly or used interchangeably over time. This reinforces the recommendation, stated above, that a single consistent ESR measurement method be used throughout longitudinal monitoring in these conditions.

From a clinical and laboratory practice perspective, the present findings support the use of the Vision C analyzer as a reliable automated ESR measurement system for routine laboratory workflows. The Vision C analyzer met RCPA allowable total error criteria across all evaluated measurement ranges, including the high-ESR subgroup (>80 mm/h), where the mean bias of −6.99 mm/h represents approximately 6–9% of the measurement range—well within the ±30% TEa threshold. The operational advantages of the Vision C analyzer—including shorter turnaround time, closed-tube sampling, and reduced operator dependency—make it a practical choice for high-volume clinical laboratories [8,9,17]. Nevertheless, the wide limits of agreement observed in the high-ESR subgroup (−30.75 to 16.77 mm/h, spanning approximately 47 mm/h) and the limited sample size in this range (*n* = 20) introduce uncertainty that warrants interpretive caution when individual high-ESR results are used to guide time-sensitive clinical decisions. This caveat should not be read as a recommendation for routine manual confirmation but rather as a call for larger, dedicated studies to characterise inter-method agreement more precisely at elevated ESR levels. Furthermore, when transitioning between ESR measurement methods in longitudinal patient follow-up, clinicians should be aware that systematic differences between automated and reference methods may result in apparent changes in ESR that reflect methodological rather than true biological variation. Laboratory reports should therefore clearly indicate the method used for ESR measurement to facilitate accurate clinical interpretation.

Given that the observed constant bias is substantially attributable to anticoagulant-related methodological differences (EDTA versus sodium citrate) rather than to intrinsic inaccuracy of the automated optical measurement principle, the two methods should not be regarded as numerically interchangeable, and method-specific reference intervals—rather than a single shared reference range—may be warranted when reporting and interpreting ESR results generated by EDTA-based automated analyzers versus the citrate-based Westergren reference method.

The present study has several limitations that should be acknowledged. First, the study was conducted at a single center, which may limit the generalizability of the findings to other laboratory settings, patient populations, or instrument configurations. Second, the high-ESR subgroup (>80 mm/h) comprised only 20 samples, 20/228, 8.8% of the total cohort), which restricted the statistical power of subgroup-specific analyses and resulted in extremely wide confidence intervals for the estimated mean bias and limits of agreement in this range; given this limited sample size, these sub-group-specific findings should be interpreted with caution and explicitly require confirmation in larger cohorts. Third, although patient hematocrit (HCT) values were available from concurrent complete blood counts for all participants, the manufacturer-recommended manual HCT-based calibration was not applied to the Vision C results used in this study, and dedicated interference studies evaluating the potential impact of hematocrit, hemolysis, or hyperfibrinogenemia on inter-method agreement were not performed; as ESR is known to be influenced by hematocrit and plasma protein composition, uncorrected hematocrit effects may have contributed to the observed bias, particularly in anemic patients or at elevated ESR levels [1]. This concern is corroborated by direct comparisons of automated EDTA-based ESR measurements against hematocrit-corrected Westergren values, which have demonstrated progressively widening discordance with decreasing hematocrit and increasing ESR [31], as well as by hematocrit- and MCV-adjusted aggregation-index models developed specifically to account for this confounding effect [32]. Fourth, the extrapolation of 20 min kinetic sedimentation data to an equivalent 60 min Westergren value by the Vision C analyzer introduces an inherent source of systematic error that may account, at least in part, for the constant bias identified in the present study [11]. Fifth, internal quality control (IQC) for the Vision C analyzer was performed exclusively with manufacturer-provided ready-to-use ball-bearing control materials at two distinct levels; although these controls verify the mechanical and optical function of the analyzer, they are non-biological in nature and do not replicate the rheological properties of native erythrocyte suspensions. Conventional multi-level blood-based ESR controls were not used, and this IQC approach should therefore be regarded as an additional limitation, since matrix-related sources of analytical variability may not be fully captured. Sixth, the present study did not include replicate measurements by the manual Westergren method; consequently, the within-method imprecision of the reference procedure could not be estimated. The manual Westergren method is known to carry a coefficient of variation (CV) of approximately 10–20% [19], meaning that a proportion of the observed inter-method differences may reflect inherent Westergren irreproducibility rather than Vision C inaccuracy. The consistent negative bias observed in the present study should therefore be interpreted as a between-method difference rather than a unidirectional measurement error attributable solely to the automated system. Seventh, the present TEa assessment was conducted at the level of aggregate (subgroup-mean) bias rather than individual-patient pass/fail classification; future studies with larger sample sizes could apply a categorical framework—classifying each patient’s result as meeting or failing the applicable TEa criterion and comparing pass rates across the three clinical ESR subgroups (e.g., via chi-square or Fisher’s exact test)—to provide a more granular, patient-level assessment of analytical acceptability. Eighth, the original a priori sample size calculation was based on statistical power for Pearson correlation rather than on the precision of the bias and limits-of-agreement estimates recommended by CLSI EP09-ED3 for method-comparison studies; consequently, subgroup-level precision was not explicitly planned in advance, and the comparatively wide confidence intervals observed in the intermediate- and high-ESR subgroups (*n* = 36 and *n* = 20, respectively) reflect this design limitation. Future studies should determine sample size prospectively based on the desired half-width of the bias confidence interval within each clinically relevant subgroup. Finally, as noted in prior multicenter evaluations of automated ESR systems [9], the use of different anticoagulants across methods—EDTA for the Vision C analyzer and sodium citrate for the Westergren reference—represents a fundamental methodological difference that precludes direct sample-matched comparisons and may independently contribute to systematic inter-method discrepancies. Despite these limitations, the present study provides clinically relevant, guideline-based method comparison data for the Vision C analyzer that may inform laboratory validation processes and support evidence-based decisions regarding its clinical implementation.

## 5. Conclusions

In conclusion, the Vision C automated ESR analyzer showed a statistically significant, level-dependent negative bias relative to the manual Westergren reference method, with limits of agreement widening progressively—and bias magnitude increasing—at higher ESR levels; these findings are based primarily on Bland–Altman-derived parameters, as Passing–Bablok regression was applied to the full sample only. As the observed differences incorporate both the Vision C measurement error and inherent Westergren irreproducibility, this bias should be interpreted as a between-method difference rather than a unidirectional inaccuracy of the automated system. Despite this bias, the analyzer demonstrated strong overall correlation with the Westergren method and met the RCPA allowable total error criteria across the full clinical measurement range, including the high-ESR subgroup (>80 mm/h), supporting its suitability for routine laboratory use. Nonetheless, the wide limits of agreement in the high ESR range (−30.75 to 16.77 mm/h) and the limited sample size in this subgroup (*n* = 20) introduce uncertainty that warrants interpretive caution when individual results are used to guide time-sensitive clinical decisions; this should not, however, be read as a recommendation for routine manual confirmation, as the method meets its predefined acceptance criteria. Larger dedicated studies in the high ESR range are needed to characterise inter-method agreement more precisely. Method-specific reporting and consistent use of the same measurement system in longitudinal patient follow-up are strongly recommended to ensure accurate clinical interpretation.

## Figures and Tables

**Table 1:** Descriptive characteristics of the study population (*n* = 228).

Characteristic	Value	Range/IQR
Total patients, *n*	228	—
Sex, Female/Male, *n* (%)	138/90	60.5%/39.5%
Age, years, median (IQR)	56	43.8–65.0 (range: 18–84)
Vision C ESR, mm/h, median (IQR)	15	10.0–35.0 (range: 5–115)
Westergren ESR, mm/h, median (IQR)	11	4.0–32.2 (range: 2–119)
ESR < 40 mm/h, *n* (%)	172 (75.4%)	—
ESR 40–80 mm/h, *n* (%)	36 (15.8%)	—
ESR > 80 mm/h, *n* (%)	20 (8.8%)	—

ESR: erythrocyte sedimentation rate; IQR: interquartile range.

**Table 2:** Bland–Altman and Passing–Bablok regression parameters by ESR subgroup (Westergren vs. Vision C).

ESR Subgroup	*n*	Mean Bias, mm/h (95% CI) †	95% Limits of Agreement	r	PB Regression Equation
Overall	228	−3.30 (−4.25 to −2.35) *	−17.65 to 11.06	0.961	y = −2.857 + 0.971x
<40 mm/h	172	−2.98 (−3.76 to −2.21) *	−13.15 to 7.18	0.821	y = −3 + 1.0x
40–80 mm/h	36	−2.75 (−6.35 to 0.85)	−24.33 to 18.83	0.740	Not performed ‡
>80 mm/h	20	−6.99 (−12.31 to −1.68) *	−30.75 to 16.77	0.687	Not performed ‡

Difference direction: Vision C. − Westergren. Negative values indicate lower ESR results with Vision C compared with the Westergren reference method. * Statistically significant bias (95% CI excludes zero). † Negative bias indicates Vision C reports lower values than Westergren. PB: Passing–Bablok; CI: confidence interval; LoA: limits of agreement; r: Pearson correlation coefficient. ‡ Within-subgroup Passing–Bablok regression was not performed due to truncated measurement range.

**Table 3:** Correspondence between clinical ESR subgroups and TEa assessment strata.

Clinical Subgroup	*n*	TEa Stratum	TEa Criterion Applied
<40 mm/h (ESR ≤20)	143	≤20 mm/h	Absolute bias, ±6.0 mm/h
<40 mm/h (ESR 20–40)	29	>20 mm/h	Percentage bias, ±30%
40–80 mm/h	36	>20 mm/h	Percentage bias, ±30%
>80 mm/h	20	>20 mm/h	Percentage bias, ±30%

ESR: erythrocyte sedimentation rate; TEa: allowable total error. The ≤20 mm/h and >20 mm/h strata correspond to the two RCPA acceptance criteria (absolute vs. percentage bias) and are nested within, rather than independent of, the clinical subgroups used elsewhere in this manuscript.

## Data Availability

The data presented in this study are available upon reasonable request from the corresponding author.

## Abbreviations

The following abbreviations are used in this manuscript:

ESR	Erythrocyte Sedimentation Rate
ICSH	International Council for Standardization in Haematology
CLSI	Clinical and Laboratory Standards Institute
EDTA	Ethylenediaminetetraacetic Acid
RCPA	Royal College of Pathologists of Australasia
IQR	Interquartile Range
LoA	Limits of Agreement
CI	Confidence Interval
TEa	Allowable Total Error
DAS28	Disease Activity Score 28
RA	Rheumatoid Arthritis
ACR	American College of Rheumatology
EULAR	European League Against Rheumatism
IQC	Internal Quality Control
AAB MLE	American Association of Bioanalysts Molecular Laboratory Evaluation
PB	Passing–Bablok
r	Pearson Correlation Coefficient
SD	Standard Deviation
